# The Role of Natural Fibers in the Building Industry—The Perspective of Sustainable Development

**DOI:** 10.3390/ma18163803

**Published:** 2025-08-13

**Authors:** Agnieszka Przybek

**Affiliations:** 1 CUT Doctoral School, Cracow University of Technology, Warszawska 24, 31-155 Cracow, Poland; agnieszka.przybek@pk.edu.pl; 2 Faculty of Material Engineering and Physics, Cracow University of Technology, Jana Pawła II 37, 31-864 Cracow, Poland; 3 Interdisciplinary Center for Circular Economy, Cracow University of Technology, Warszawska 24, 31-155 Cracow, Poland

**Keywords:** natural fibers, modern materials, sustainable building, CO_2_ reduction, revolution in the world

## Abstract

Contemporary construction faces the need to reduce its negative impact on the environment, prompting designers, investors, and contractors to seek more sustainable materials and technologies. One area of dynamic development is the use of natural fibers as an alternative to conventional, often synthetic, building components. Plant- and animal-based fibers, such as hemp, flax, jute, straw, bamboo, and sheep’s wool, are characterized by low energy consumption in production, renewability, and biodegradability. Their use is in line with the concept of a circular economy and reduces the carbon footprint of buildings. Natural fibers offer a number of beneficial physical and functional properties, including good thermal and acoustic insulation parameters, as well as hygroscopicity, which allows for the regulation of indoor humidity, improving air quality and comfort of use. In recent years, there has also been a renaissance of traditional building techniques, such as straw construction, often combined with modern engineering standards. Their potential is particularly recognized in green and energy-efficient construction. The article provides an overview of the types of natural fibers available for use in construction and analyzes their technical, environmental, and economic properties. It also draws attention to current regulations, standards, and certifications (e.g., LEED, BREEAM) that promote the popularization of these solutions. In light of the analyzed data, the role of natural fibers as a viable alternative supporting the transformation of the construction sector towards sustainable development is considered.

## 1. Introduction

The partial shift away from the utilization of natural materials in construction, despite their numerous advantages, can be attributed to several interrelated factors. One of the primary reasons is the industrialization of the construction sector, which favors the use of synthetic materials, such as concrete and steel. Contemporary buildings predominantly employ reinforced concrete, steel, or masonry structures. While many detached houses are constructed of wood, dwellings built of engineered materials are significantly more prevalent. These materials, although often more durable and amenable to mass production, incur a substantial environmental cost. For instance, traditional building materials such as concrete consume considerable energy and natural resources, contributing to approximately 40% of resource extraction and 45–65% of landfill waste in industrialized countries [1,2,3,4,5]. The environmental impact of these materials has led to an increasing awareness and demand for sustainable alternatives. However, transformation has been gradual due to entrenched practices and economic considerations [6,7,8,9].

Moreover, the perception that natural materials exhibit inferior reliability or durability compared to their synthetic counterparts impedes their widespread adoption. Natural materials such as wood and straw may be susceptible to moisture and pest infestation, raising concerns regarding their longevity and structural integrity [10,11,12].

Moreover, economic factors play a significant role in this transformation. The construction industry frequently prioritizes cost efficiency and productivity, resulting in a preference for materials that can be produced and assembled expeditiously. Natural materials typically necessitate more labor-intensive harvesting and preparation processes, which can increase costs and project durations. Additionally, the absence of standardized practices and regulations concerning the utilization of natural materials can engender uncertainty among builders and developers, further dissuading their implementation [13,14,15,16].

Contemporary cultural and aesthetic preferences in modern housing construction have shifted towards a more industrial aesthetic, which often prioritizes the utilization of concrete, glass, and steel over traditional natural materials. This trend is further reinforced by the marketing and branding strategies that position modern buildings as symbols of progress and innovation, potentially overshadowing the advantages of employing sustainable, natural materials [17].

While natural materials offer numerous benefits, including sustainability and reduced environmental impact, the construction industry based on synthetic materials is driven by economic, practical, and cultural factors. The challenge persists in effectively integrating these materials into contemporary building practices, balancing the imperatives of sustainability with the requirements of modern building standards.

This review article significantly builds on existing research by offering a new perspective on using natural fibers as a potential alternative to common insulation materials. The review presents a comprehensive comparison of the properties of natural fibers with popular insulation materials, considering both their thermal and acoustic performance and environmental aspects. This approach allows for a more accurate assessment of their potential and limitations. The research discusses in detail the environmental benefits of natural fibers, such as a lower carbon footprint, biodegradability, and the possibility of using them as agricultural waste. Highlighting these aspects makes the article an important contribution to the sustainability discussion. The article not only points out the advantages of natural fibers but also takes an in-depth look at existing obstacles, such as production costs, moisture resistance, or durability, while offering suggestions for potential solutions. The review opens up new research horizons, suggesting areas for further exploration, such as innovative methods for modifying fibers, their hybrid use with other materials, and research into their long-term use. The inclusion of various perspectives—from engineering and technology to economic and social—gives the article a unique interdisciplinary character. By critically evaluating the existing literature and providing new findings, this review makes an important contribution to the discussion on sustainability and innovative applications of natural fibers.

The selected research had to focus on the insulating properties of natural fibers and their potential for use as an alternative to conventional insulation materials, such as polystyrene, mineral wool, or polyurethane foam. Priority was given to studies using robust research methods, including experimental analyses of the thermal, acoustic, and mechanical properties of natural fibers. Work with unclear methodology or insufficient data documentation was excluded. The focus was mainly on publications from the last 5 years to include the latest technological developments and innovations in sustainable materials. Studies from different regions of the world were included to provide a global perspective and diversity of natural materials used in different countries. Priority was given to papers discussing the environmental benefits and economic viability of natural fibers in the context of their commercialization. Studies integrating knowledge from different disciplines, such as materials engineering, chemistry, ecology, and economics, were selected to provide a comprehensive view of the topic. It was assumed that the properties of natural fibers studied in different regions of the world are sufficiently universal to be compared and generalized. The article assumes that the pursuit of sustainability and the reduction of greenhouse gas emissions will be a key motivating factor for the replacement of conventional insulation materials. It is assumed that technological innovations will enable the future scaling of natural fiber production for the mass market. However, there is a limited number of studies that evaluate the durability and long-term behavior of natural fibers, which may affect the reliability of the conclusions. The variety of approaches to measuring insulating properties can make it difficult to compare results from different studies. Some natural fibers may only be available in specific regions, limiting their potential versatility. Many studies omit detailed economic analyses of processing and large-scale production costs. Unfortunately, there is limited research that considers the effects of variables such as moisture, mold, or insects on the performance of natural fibers in practical applications. Consideration of these assumptions and limitations allows for a more critical view of the results of the reviewed studies and highlights the need for further exploration of the topic.

## 2. A Historical Overview of Pre-Industrial Revolution Housing Construction Methods

Before the onset of the Industrial Revolution, residential construction was significantly influenced by the regional availability of materials and the prevailing environmental conditions. Various ancient civilizations utilized a diverse range of materials, including bamboo, earth, and wood, to construct dwellings that were not only functional but also adapted to their specific climatic conditions and cultural practices.

Bamboo served as a significant construction material in numerous ancient civilizations, particularly in Southeast Asia. It was favored for its lightweight yet robust characteristics, rendering it an optimal selection for residential structures. Ancient societies frequently utilized bamboo as the primary component for house construction, owing to its abundance and capacity to provide adequate protection from environmental elements [18,19,20,21]. This practice exemplifies the resourcefulness of early societies in utilizing indigenous flora for shelter.

Apart from bamboo, earth has been the primary construction material throughout history. Archaeological evidence indicates that raw earth was utilized extensively in ancient structures, with some of the oldest known earth-based dwellings dating from approximately 6000 BC—Jericho in Israel [22,23,24]. The utilization of rammed earth and adobe techniques facilitated the construction of durable dwellings capable of withstanding various environmental stresses. Walls of substantial thickness composed of earth not only provided structural support but also contributed to thermal regulation by moderating interior temperature [25].

Wood constituted another critical material for residential construction, particularly in regions with abundant forest resources [26,27,28,29,30,31,32]. In areas such as Kashmir, traditional wooden dwellings exemplify the cultural heritage and architectural styles of the region, demonstrating the adaptability of local materials to the inhabitants’ requirements [33,34,35]. The utilization of wood not only provided structural integrity but also facilitated intricate designs and craftsmanship that reflected the cultural identity of the builders.

**Figure 1:**
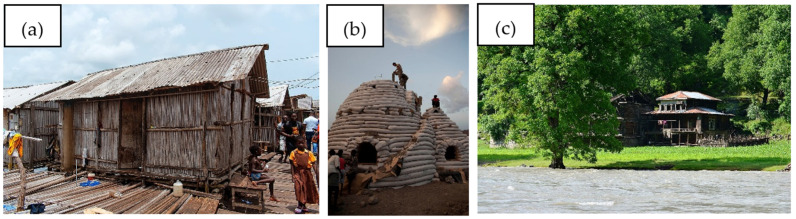
(**a**) Bamboo house showcasing traditional construction methods in Southeast Asia, emphasizing the use of local materials and sustainable techniques (adapted from [36]), (**b**) SuperAdobe dome construction in a local village (adapted from [37]), (**c**) Kashmir’s house lying on the border between India and Pakistan separated from the plains of India by the Pir Panjal mountain range (adapted from [38]).

Bamboo, earth, and wood constituted the primary materials utilized, each contributing to the functionality and durability of ancient dwellings. Figure 1 illustrates examples of the aforementioned structures.

Construction, as one of the oldest fields of human activity, has developed in various cultures and climates, which has resulted in a huge variety of construction methods and materials used. The literature on the subject describes many traditional and modern techniques for erecting buildings, which can be classified according to the materials used and the technology employed. A detailed overview of the most important construction methods used around the world, based on historical data and contemporary sources, is presented below.

### 2.1. Wooden Construction

Wooden construction is one of the oldest methods of erecting residential structures. Wood as a construction material is characterized by a favorable strength-to-weight ratio and ease of processing, which allows for the creation of both structural frames and solid log walls. This technology has been and continues to be widely used in temperate and cold climates, particularly in Northern Europe, North America, and Siberia [39,40]. The main disadvantages include wood’s susceptibility to moisture and fire, which requires proper maintenance.

### 2.2. Masonry Construction

Brick and stone masonry has been the basic technique in many regions of the world for centuries. These materials are characterized by high durability and good thermal insulation, which makes them suitable for various climatic conditions. Masonry methods were developed intensively in the European area [41,42].

### 2.3. Earth Construction

Earth construction includes techniques such as adobe, cob, and rammed earth, where earth and clay are the primary building materials. These methods are characterized by low costs and high environmental friendliness, as well as good insulating properties. They have been known for millennia and are still used today in Latin America, Africa, Asia, and some parts of Europe [43,44]. The disadvantage is their susceptibility to erosion and degradation under the influence of water, which requires proper maintenance and finishing.

### 2.4. Concrete and Reinforced Concrete Construction

Concrete and reinforced concrete are currently among the most widely used building materials in the world. Their mechanical properties enable the construction of structures with high strength and durability, including multi-story buildings and infrastructure. This method has been developing particularly intensively since the 20th century due to the possibility of prefabrication and mass production of structural elements [45,46]. However, high CO_2_ emissions during cement production and high weight are significant environmental challenges.

### 2.5. Construction with Natural and Renewable Materials

Growing interest in ecology and sustainable development has led to the popularization of construction based on materials such as straw, bamboo, and hemp. These materials are characterized by low weight, good insulating properties, and a low ecological footprint. They are traditionally used in many regions of the world, especially in Asia, South America, and Europe [47,48]. However, adequate protection against moisture and fire is necessary.

### 2.6. Prefabricated and Modular Construction

Prefabrication and modular construction techniques enable rapid building construction through the assembly of factory-produced components. These solutions have gained popularity, especially in countries with a high level of industrialization and technological development, such as Japan, Sweden, and the USA [49]. This method allows for increased efficiency and quality control, although it limits the possibility of individualizing projects.

### 2.7. Stone and Megalithic Construction

Large stone structures, erected without the use of mortar or with minimal use of it, are an example of ancient construction technologies. Their durability and monumentality are well-documented in cultures such as the Egyptian, Inca, and megalithic Europe [50,51]. Currently, this technique is not used in modern residential construction, but it is an important part of cultural heritage.

### 2.8. Clay and Wattle, and Daub Construction

The combination of clay, straw, and wooden wattle was a common method in many cultures, especially in Europe and Africa. The wattle and daub technique allowed for the quick and inexpensive construction of walls with good thermal insulation, although it required regular maintenance and protection against moisture [52]. Table 1 presents a comparison of house construction methods, taking into account the main building materials, geographical range, advantages, and disadvantages, as well as historical and modern examples.

## 3. Today’s Use of Traditional Housing Construction Practices

Residential structures constructed from natural materials have been a significant aspect of traditional housing construction across diverse cultures, reflecting the intricate relationship between human habitation and the surrounding environment. The utilization of locally sourced materials not only enhances the aesthetic qualities of these structures but also contributes to their sustainability and energy efficiency.

A notable exemplar is the Bosnian house Chardaklia, which employs traditional construction methodologies that incorporate materials from the immediate natural environment. This approach establishes a symbiotic relationship between the dwelling and its surroundings, resulting in a structure that manifests as a ‘natural man-made environment’ [53,54,55,56]. Such architectural designs prioritize thermal comfort across all seasons, optimizing the building envelope and utilizing renewable energy sources, such as firewood and beeswax candles, before the advent of electricity [57,58,59,60].

Furthermore, the environmental impact of dwellings constructed from natural materials is notably advantageous. A life cycle assessment (LCA) study of single-family residences in Eastern Slovakia revealed that those constructed with natural materials, such as clay, straw, and wood, exhibited negative CO2-equivalent emissions during the use phase, in stark contrast to conventional materials such as aerated concrete [61,62]. This finding underscores the ecological benefits of utilizing natural materials, which not only mitigate greenhouse gas emissions but also foster a more salubrious living environment.

**Figure 2:**
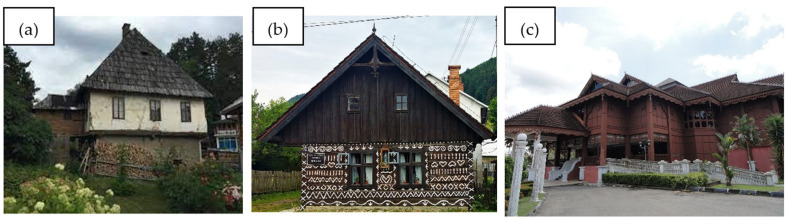
(**a**) Bosnian house Chardaklia (adapted from [59]), (**b**) Eastern Slovakia house (adapted from [70]), (**c**) Malay house (adapted from [71]).

The thermal performance of traditional houses is a significant aspect influenced by the selection of construction materials. For instance, traditional houses in Malaysia are designed to optimize natural ventilation and thermal comfort, utilizing lightweight materials that effectively dissipate heat during nighttime hours [63,64,65]. Day and night thermal comfort in residential buildings in Malaysia is a complex issue influenced by multiple factors, including building design, orientation, ventilation strategies, and construction materials. Considering Malaysia’s hot and humid climate, achieving thermal comfort while minimizing energy consumption is essential for sustainable living [66,67,68,69]. Figure 2 illustrates the aforementioned houses.

Nevertheless, there existed a concerning trend toward the substitution of traditional materials with modern manufacturing alternatives, which potentially compromised the environmental performance of a building. Research indicated that this transition not only increased energy consumption but also diminished the thermal comfort inherently provided by traditional materials [69]. The shift from natural to modern materials frequently disregards the sustainable practices embedded in traditional housing construction that have been refined over centuries to address both environmental and social requirements [72].

Dwellings constructed from natural materials exemplify a sustainable approach to residential construction that aligns with environmental principles. These structures offer substantial ecological advantages, including reduced carbon emissions and enhanced thermal comfort, while simultaneously preserving cultural heritage and local craftsmanship. The contemporary trend towards modern construction materials challenges these traditional practices, underscoring the necessity for a reevaluation of natural building methods in present-day residential construction. Given the multifaceted benefits of dwellings constructed from natural materials, it is pertinent to examine the factors that have led to the decline of this practice.

## 4. Returning to Traditional Materials: The Utilization of Natural Fibers in Construction

**Figure 3:**
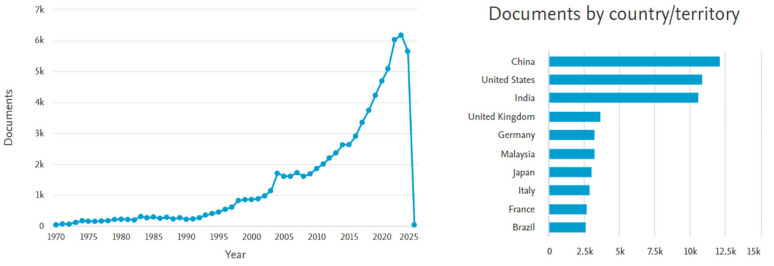
Publications on natural fibers in recent years and a list of publications by country/region [73].

A search engine query in the Scopus database was conducted to assess researchers’ interest in natural fibers. Upon entering the keyword ‘natural fibers’, the results obtained are presented in Figure 3 [73]. The data indicate a substantial increase in scholarly attention to the topic of natural fibers in recent years. The highest number of publications on this subject originated from China, the United States, and India.

The resurgence of natural fibers in construction reflects an increasing recognition of their environmental benefits and mechanical properties. Natural fibers such as jute, hemp, and sisal are increasingly utilized as reinforcing materials in composite structures due to their low density, cost-effectiveness, and biodegradability [74,75,76]. These fibers present a sustainable alternative to synthetic fibers, which often necessitate energy-intensive manufacturing processes and contribute to environmental degradation [77,78]. The incorporation of natural fibers not only reduces the carbon footprint of building materials but also enhances the overall sustainability of construction practices [76].

Research indicates that natural fiber composites can achieve mechanical properties comparable to traditional materials. For instance, studies have demonstrated that natural fibers can effectively substitute glass fibers in composite materials, providing similar strength and stiffness while exhibiting significantly lower density [79,80]. The incorporation of natural fibers into building materials such as cement and plaster has been shown to enhance thermal properties, acoustic properties, and hydrothermal comfort and reduce density, resulting in lighter and more energy-efficient end products [81,82].

**Figure 4:**
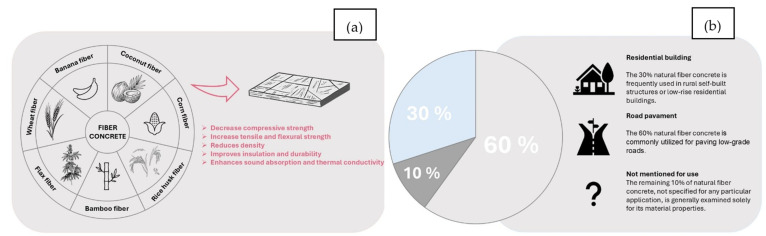
(**a**) Performance evaluation of fibers in cementitious composites. (**b**) Application examples (based on [94]).

Furthermore, the use of natural fibers in cementitious matrices has been investigated for their potential in reinforcing masonry structures, demonstrating their versatility in various building applications [83]. Natural fibers have garnered considerable attention in the field of cementitious composites due to their potential to enhance mechanical properties while promoting sustainability. The incorporation of natural fibers, such as jute, bamboo, and palm fibers, into cement matrices can improve the mechanical performance of these composites, particularly in terms of tensile strength, ductility, and toughness. This improvement is primarily attributed to the fibers’ capacity to bridge cracks and mitigate crack propagation within the brittle cement matrix [84,85]. One of the critical factors influencing the performance of natural fibers in cementitious composites is their interaction with the alkaline environment of the cement matrix. The high alkalinity, resulting from the hydration of cement, can adversely affect the durability and mechanical properties of natural fibers. For instance, studies have demonstrated that the treatment of fibers can significantly enhance their resistance to alkaline degradation, thereby improving the overall durability of the composite [86,87]. However, surface modifications, such as the application of coatings or chemical treatments, can improve the fiber’s surface properties, enhancing the adsorption of calcium ions and improving the fiber–matrix bond strength [88,89]. Moreover, the geometry and loading of natural fibers play an essential role in determining the mechanical properties of the resulting composites. Research indicates that the arrangement and aspect of fibers can influence the composite’s compressive and flexural strengths. For example, bamboo fibers have been shown to effectively increase the uniaxial compressive strength of cement composites due to their unique geometry and interlocking capabilities [90]. Additionally, the utilization of aligned fibers can further enhance the mechanical performance by optimizing the load transfer mechanisms within the composite [91]. The sustainability aspect of employing natural fibers in cementitious composites warrants consideration. Natural fibers are biodegradable, lightweight, and mostly sourced from renewable resources, rendering them an environmentally favorable alternative to synthetic fibers. Their incorporation into construction materials contributes to the reduction of environmental degradation and the promotion of sustainable building practices. Moreover, the utilization of natural fibers can result in cost-effective solutions in construction, as they are typically less expensive than synthetic alternatives [92,93]. The integration of natural fibers into cementitious matrices presents a promising avenue for enhancing the mechanical properties of construction materials while promoting sustainability. The challenges posed by the alkaline environment of cement can be mitigated through effective fiber treatment and surface modifications, while the geometry and loading of fibers can be optimized to maximize their reinforcing capabilities. As research continues to explore the potential of various natural fibers, their application in cementitious composites is likely to expand, contributing to more sustainable construction practices. Figure 4a,b illustrate the performance evaluation of fibers in composites and the application examples.

The utilization of natural fibers in construction represents a significant shift toward more sustainable building practices. Their mechanical properties, environmental benefits, and economic advantages render them an attractive option for various applications in the construction industry. As research progresses, the potential for natural fibers to play an essential role in the future of sustainable construction appears promising. However, this assertion warrants further investigation.

Natural fibers are increasingly used in construction due to their mechanical properties, availability, low weight, and low energy consumption during production. Their use in composite materials—both polymeric and mineral—promotes the development of low-emission technologies. Below is a classification of the most popular natural fibers, methods of their processing, and examples of the construction composites in which they are used.

### 4.1. Types of Natural Fibers Used in Construction

Natural fibers are divided into three main groups based on their origin: plant, animal, and mineral. In construction, plant fibers are most commonly used, mainly derived from the stems, leaves, seeds, or fruits of cultivated plants. Plant fibers are most often used in construction composites due to their availability, low weight, and good adhesion to many matrices.

#### 4.1.1. Plant Fibers

##### Flax (*Linum usitatissimum*)

Flax fibers come from the stems of the plant. They consist mainly of cellulose (60–80%), hemicellulose (10–20%), and lignin (2–5%). They are characterized by very high tensile strength (500–900 MPa) and a modulus of elasticity reaching 70 GPa, which makes them competitive with synthetic fibers. Thanks to their good adhesion to resins and low density (approx. 1.5 g/cm^3^), they are used, among other things, in facade panels and bio-composite prefabricated elements [95,96].

##### Hemp (*Cannabis sativa*)

Hemp fibers, obtained from stems, are rich in cellulose and have natural resistance to fungi, mold, and moisture. Their tensile strength reaches 900 MPa, and their modulus of elasticity is approximately 60 GPa. It is widely used in thermal insulation materials, structural panels, and cement composites as microscopic reinforcement [95,97]. In addition, it is also used in historical construction (e.g., mortar from Ellora Cave in India) as a natural filler in lime mortar (yang hemp fiber in lime mortar) [98].

##### Jute (*Corchorus capsularis*)

This is one of the most commonly used fibers in developing countries. Jute is characterized by lower strength (400–800 MPa) and a Young’s modulus of approximately 30 GPa, but this is compensated for by its very low price and ease of acquisition. It is mainly used as reinforcement in clay and gypsum plasters, finishing materials, and acoustic panels [93,99]. Jute often appears in cement composites and as a dyed nonwoven fabric in green buildings.

##### Sisal (*Agave sisalana*)

Obtained from the leaves of the sisal agave, it contains a lot of lignin (over 10%), which gives it greater stiffness than other fibers. It is characterized by good adhesion to polymer matrices, and its tensile strength ranges from 400 to 700 MPa. It is mainly used in thermoformed composites for the production of doors, wall panels, and cladding [95].

##### Coconut (*Cocos nucifera*)

Coconut fibers come from the mesocarp of the fruit and contain a lot of lignin (~45%), which gives them flexibility and significant energy absorption capacity. They are less mechanically resistant (100–200 MPa), but their acoustic and thermal insulation properties make them an excellent material for fillings, insulation, and vibration-damping panels [100,101].

##### Cotton (*Gossypium* spp.)

Fibers are derived from plant seeds. They consist almost exclusively of cellulose (over 90%). They are characterized by good elasticity and softness, with a strength ranging from 287 to 597 MPa and a Young’s modulus in the range of 5.5–12.6 GPa. Although less durable than stem fibers, cotton is used in insulation materials (biofelt, nonwovens) and thin-layer composites based on bio-resins and starch [93,100].

#### 4.1.2. Animal and Mineral Fibers

##### Sheep Wool

Wool is a natural protein fiber with excellent insulating properties—both thermal and acoustic. The density of wool is approximately 1.3 g/cm^3^, and its thermal conductivity ranges from 0.035 to 0.050 W/m·K. Wool is hygroscopic and self-extinguishing, which makes it a safe and environmentally friendly insulating material. In construction, it is mainly used in the form of mats or boards as an alternative to mineral wool [102,103].

##### Silk (*Bombyx mori*) and Horsehair

Silk and horsehair are less commonly used protein fibers. Silk has historically been used as a reinforcing fiber in Japanese and clay plasters due to its ductility and resistance to cracking. Horsehair, on the other hand, has been used to reinforce lime and gypsum mortars, especially in conservation crafts and traditional rural construction [103].

##### Asbestos (Now Banned)

**Figure 5:**
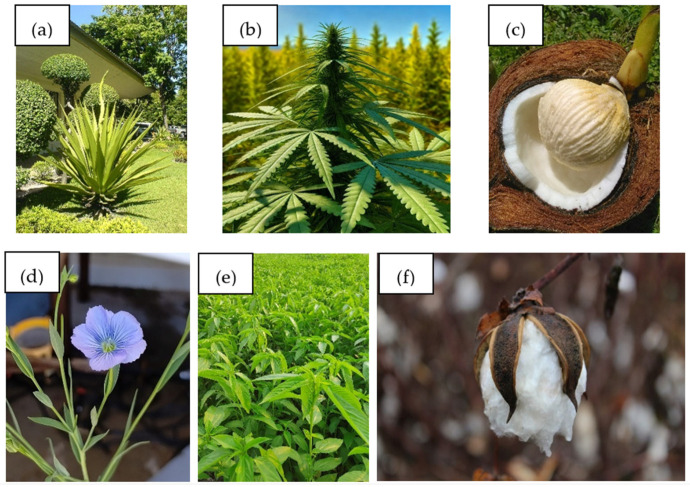
Natural plant fibers: (**a**) sizal (adapted from [104]), (**b**) hemp (adapted from [105]), (**c**) coconut (adapted from [106]), (**d**) linen (adapted from [107]), (**e**) jute (adapted from [108]), (**f**) cotton (adapted from [109]).

Asbestos is a naturally occurring mineral fiber, widely used in the 20th century due to its resistance to fire, acids, and high temperatures. It was used in roofing tiles, pipes, fireproof materials, and insulation. Due to its strong carcinogenic properties, it has been banned in most countries (in the EU since 2005) and replaced with ceramic, glass, and plant fibers [101]. Figure 5 shows photographs of natural plant fibers.

### 4.2. Methods of Processing Natural Fibers

Natural fibers require modification to ensure durability and good adhesion to matrices (especially mineral and polymer matrices). The most commonly used treatment methods are presented below. Various physical, chemical, and biological methods are used to improve the compatibility of natural fibers with the composite matrix and increase their durability in a humid environment. Table 2 presents a comparison of natural fiber processing methods.

#### 4.2.1. Physical Processing [110,111]

- Mechanization (cutting, grinding): Reduces the length of fibers to the desired fraction;
- Thermal modification: Includes drying or steam treatment to remove moisture;
- Ultrasound: Improves the delamination and dispersion of fibers in the matrix.

#### 4.2.2. Chemical Processing [112,113,114]

- Alkalization (NaOH): Removes lignin and hemicellulose, increasing the contact surface with the matrix;
- Acetylation: Reduces the hygroscopicity of the fibers;
- Silanization: Improves adhesion between the fiber and epoxy resin or cement.

#### 4.2.3. Biological Treatment [115,116]

- Enzymatic fermentation: Reduces the content of undesirable substances, e.g., pectins;
- Cellulolytic bacteria: Reduce the non-adhesive parts of the fibers.

### 4.3. Natural Fiber Composites in Construction

Natural fibers are used as reinforcement in composites, both polymer and cement, as well as in insulation materials. Depending on the type of matrix, several classes of applications can be distinguished. Table 3 presents examples of composites with natural fibers, along with their corresponding parameters.

#### 4.3.1. Polymer Composites [117,118]

- Matrices: Epoxy resins, polyester resins, polylactide (PLA);
- Applications: Facades, wall panels, furniture, roofing;
- Advantages: Lightweight, biodegradable, low CO_2_ emissions.

#### 4.3.2. Cement Composites [119,120]

- Matrices: Portland cement, geopolymers;
- Applications: Wall blocks, plasters, facade panels;
- Advantages: Improved flexural strength, better crack resistance.

#### 4.3.3. Insulation Composites [121,122]

- Matrices: Natural binders (starch, lignin), bio-resins;
- Applications: Insulation boards, acoustic mats;
- Advantages: High water vapor sorption, microclimate regulation.

## 5. What Factors Underlie the Recent Increase in Popularity of Natural Fibers?

The increasing prevalence of natural fibers in contemporary applications can be attributed to a convergence of environmental, economic, and performance factors. As industries progressively emphasize sustainability, natural fibers have emerged as a viable alternative to synthetic materials, particularly in composite applications. This transition is predominantly due to an enhanced awareness of the environmental implications associated with conventional synthetic fibers, which frequently entail substantial energy consumption and carbon emissions during production [76,123]. The production of traditional synthetic fibers, including carbon, aramid, glass, and basalt fibers, is characterized by significant energy consumption and carbon dioxide (CO_2_) emissions. Each type of fiber exhibits distinct production processes that contribute to its environmental footprint. The production of carbon fibers, primarily from polyacrylonitrile (PAN), is notably energy-intensive. Studies indicate that the energy requirement for carbon fiber production can range from 330 to 500 MJ/kg, with some estimates suggesting even higher values depending on the specific processes employed [124,125]. The carbonization process, which transforms stabilized PAN fibers into carbon fibers, is particularly energy-demanding, consuming approximately 70% of the total energy used in production [124]. Consequently, the CO_2_ emissions associated with carbon fiber production are estimated to be approximately 10–20 kg of CO_2_ per kilogram of fiber produced, largely due to the combustion of the fossil fuels used in the heating processes [124,126]. The production of aramid fibers, such as Kevlar, also involves substantial energy consumption. The energy requirement for aramid fiber production is estimated to be approximately 120–150 MJ/kg, with CO_2_ emissions ranging from 6 to 10 kg of CO_2_ per kilogram of fiber produced [127]. The synthesis of aramid fibers involves complex chemical processes that contribute to their environmental impact, including the utilization of hazardous chemicals that require careful handling and disposal. Glass fiber production is another energy-intensive process, with energy consumption typically reported at approximately 20–30 MJ/kg [128]. The CO_2_ emissions from glass fiber production are estimated to be approximately 2–5 kg of CO_2_ per kilogram of fiber produced. The melting of raw materials, primarily silica sand, necessitates high temperatures, which are achieved through the combustion of fossil fuels, contributing to the overall carbon footprint of the process. Basalt fibers, derived from volcanic rock, exhibit a marginally lower energy requirement compared to glass fibers, estimated at approximately 15–25 MJ/kg. The CO_2_ emissions associated with basalt fiber production are estimated to be approximately 2–4 kg of CO_2_ per kilogram of fiber produced [128]. The production process entails melting basalt rock at high temperatures, analogous to glass fiber production, but generally requires less energy due to the inherent properties of basalt. The production of traditional synthetic fibers is characterized by high energy consumption and substantial CO_2_ emissions. Carbon fibers exhibit the highest energy intensity, followed by aramid, glass, and basalt fibers. The environmental impacts of these fibers underscore the necessity for more sustainable production methods and the exploration of alternative materials.

The cost/performance ratio of various fiber types, including carbon, aramid, glass, basalt, and natural fibers, is a critical consideration in composite material applications. Each fiber type exhibits unique mechanical properties, costs, and environmental impacts, influencing their suitability for different applications. Carbon fibers are characterized by their unique strength-to-weight ratio and stiffness, rendering them suitable for high-performance applications such as the aerospace and automotive industries. However, their high production costs limit their widespread use, particularly in cost-sensitive applications [129,130]. Conversely, aramid fibers, known for their high tensile strength and thermal stability, also present a high cost, which can be prohibitive for many applications [131]. While aramid fibers enhance impact resistance and durability in composites, their environmental impact and recyclability issues raise concerns [132]. Glass fibers, in contrast, are more economical and widely utilized in various applications, including the construction and automotive sectors. They offer adequate mechanical properties but are inferior to carbon and aramid fibers in terms of strength and stiffness [133]. Basalt fibers provide a compelling alternative, exhibiting mechanical properties that can rival those of carbon fibers while being more cost-effective and environmentally sustainable [134]. Basalt fibers are derived from natural volcanic rock, making them less energy-intensive to produce compared to carbon fibers, thus presenting a more favorable cost/performance ratio for certain applications [134]. Natural fibers, such as jute, hemp, and flax, are garnering attention due to their low cost and biodegradability. They offer moderate mechanical properties and are frequently utilized in applications where environmental sustainability is prioritized over extreme performance [132]. However, their mechanical properties are generally inferior to synthetic fibers, which limits their utilization in high-stress applications [132]. The integration of natural fibers with synthetic fibers, such as aramid or carbon, can enhance the overall performance of composites while reducing the costs and environmental impact [132]. While carbon and aramid fibers provide superior mechanical properties, their high costs and environmental concerns limit their applications. Glass fibers offer a more economical option but do not match the performance of carbon or aramid fibers. Basalt fibers emerge as a viable alternative, providing a favorable cost/performance ratio while being more environmentally sustainable. Natural fibers, while cost-effective and biodegradable, typically do not meet the performance requirements of high-stress applications, unless hybridized with synthetic fibers.

The mechanical properties of natural fibers contribute significantly to their increasing prevalence. Composites reinforced with natural fibers demonstrate superior mechanical properties, including tensile strength and impact strength, which are essential for structural and automotive components [135,136]. Research indicates that these composites can effectively substitute traditional materials, offering comparable or enhanced performance while mitigating overall environmental impact [137]. Furthermore, the facile processing and accessibility of natural fibers augment their appeal as a sustainable material [138].

Furthermore, the increasing emphasis on circular economy principles has heightened interest in natural fibers, as they are frequently derived from agricultural waste or by-products, thereby promoting resource efficiency and waste reduction [139]. The potential for integrating natural fibers into biocomposite materials aligns with the broader trend toward environmentally sustainable manufacturing practices, rendering them a subject of ongoing research and development [140].

Table 4 presents comparative data on the mechanical, thermal, and environmental properties of selected natural fibers (flax, hemp, cotton, coconut, jute, and sisal) and synthetic fibers (carbon, aramid, glass, and basalt fibers). Parameters such as tensile strength, modulus of elasticity, elongation at break, density, thermal conductivity, and degradation or melting temperature were taken into account. In addition, environmental aspects such as biodegradability, raw material renewability, the impact of the production process on the environment, and recyclability were also considered. This summary allows for a comprehensive assessment of the potential of individual fibers for engineering and construction applications, taking into account both performance parameters and sustainable development.

The increase in popularity of natural fibers can be attributed to several key factors, as supported by the references provided:

1. Environmental sustainability: Natural fibers are biodegradable, renewable, and have a lower environmental impact compared to synthetic fibers [160,161,162,163]. The growing environmental consciousness and need for sustainable development have driven the demand for natural fiber-based composites as alternatives to synthetic materials [161,162];
2. Mechanical properties: Natural fibers possess good mechanical properties, such as high specific strength and stiffness, making them suitable reinforcements in composite materials [164,165,166]. The ability to enhance the mechanical performance of composites has increased their adoption in various industries [164,166,167];
3. Cost-effectiveness: Natural fibers are generally low-cost, abundant, and have shorter processing stages compared to synthetic fibers [165,168]. This cost-effectiveness has made natural fiber-based composites an attractive option for various industries, particularly the automotive, aerospace, and civil engineering sectors [168,169];
4. Lightweight and density: Natural fibers have a lower density compared to synthetic fibers, which contributes to the development of lightweight composite materials [135,139]. This property is particularly beneficial for applications in the automotive and aerospace industries, where weight reduction is an essential factor [168,169];
5. Eco-friendliness and biodegradability: The biodegradability and eco-friendly nature of natural fibers have made them a preferred choice for various applications, as they can help reduce the environmental impact of synthetic materials [160,162,163,165];
6. Versatility and customizability: Natural fibers can be obtained from a wide range of sources, such as plants, animals, and agricultural waste, providing a diverse range of options for composite development [166,170]. Additionally, the chemical structure of natural fibers can be modified to enhance their properties, further increasing their versatility [165,167].

The increase in popularity of natural fibers can therefore be attributed to their environmental sustainability, favorable mechanical properties, cost-effectiveness, lightweight characteristics, eco-friendliness, and versatility, which have made them a viable alternative to synthetic fibers in various industries, particularly in the automotive, aerospace, and construction sectors [160,161,162,164,165,166,167,168,169,171,172]. As industry continues to seek sustainable alternatives to synthetic materials, natural fibers may play a significant role in the development of environmentally friendly composites for various applications. However, it is necessary to critically examine these fibers. What are the potential limitations of their utilization?

In recent years, there has been a dynamic increase in interest in natural fibers as a reinforcing material in construction composites, both polymer and cement-based, as well as a component of insulation materials. Market data and industry reports confirm this trend, pointing to the increased adoption of environmentally friendly solutions in the construction sector. According to a report by Market Research Future (2023), the global value of the natural fiber-reinforced composite market was USD 1.05 billion in 2023, and forecasts indicate growth to USD 1.8 billion by 2032, with a significant portion of this growth coming from the construction sector [173]. Similar data is presented in a market analysis published by MarketsandMarkets, indicating that construction is the second-fastest-growing sector for this type of composite, right after the automotive industry [174]. Grand View Research estimates that the total market for natural fibers (including for construction purposes) reached a value of USD 69.23 billion in 2024 and will grow to USD 94.64 billion by 2030, which translates into a compound annual growth rate (CAGR) of 5.4% [175]. These figures include fibers used for structural composites, as well as for insulation and aesthetic applications. Even more precise forecasts are provided by Mordor Intelligence, according to which global production of natural fiber composites will reach 4.73 million tons in 2025, with a projected increase to 7.12 million tons by 2030 (CAGR = 8.5%) [176]. Such significant growth is attributed, among other things, to increasing regulatory and social pressure toward the use of renewable materials in sustainable construction. Practical evidence confirming these trends can also be found in legislative and standardization activities. For example, in 2024, hemp–lime composite (also known as hempcrete) was officially approved for use in residential construction in the United States under the International Residential Code (IRC 2024), which significantly increased its market acceptance [177]. In addition to the North American market, significant momentum is also being seen in Asia, particularly in China and India, where governments are implementing policies to promote the use of natural fibers in modular construction, facade panels, and roofing. An article published in *World Construction Today* confirms that the Asia–Pacific region is leading the way in investments in building biocomposites due to its growing population, urbanization, and simultaneous climate goals [178]. The growing interest in natural fibers also stems from their unique properties, such as biodegradability, low thermal conductivity, good mechanical strength in composites, and the ability to bind CO_2_ during the growth of the plants from which the fibers are obtained. Modern research currently focuses not only on their structural application, but also on their use in smart building materials (e.g., thermal and acoustic insulation) [179].

## 6. Disadvantages and Limitations of Natural Fibers

Natural fibers have garnered considerable attention in diverse applications, owing to their environmental advantages and renewable nature. Nevertheless, they exhibit several significant limitations that constrain their extensive utilization in composite materials. A primary disadvantage is their high moisture absorption capacity, which can result in the degradation of mechanical properties in the composites they reinforce. This hydrophilic characteristic leads to poor compatibility with hydrophobic polymer matrices, frequently causing issues such as delamination and void formation at the fiber–matrix interface [10,180,181,182]. Moisture absorption not only affects structural integrity but also results in dimensional instability, which is critical in applications requiring precise tolerances [183,184,185]. Natural fibers, such as flax and hemp, present significant challenges in construction and insulation applications, primarily due to their susceptibility to moisture, mold, and degradation. These fibers are inherently hydrophilic, which results in high moisture absorption and, consequently, an elevated risk of degradation compared to synthetic insulations. Research indicates that natural fibers like coir, hemp, and jute exhibit substantial moisture sorption, which can compromise their structural integrity and performance in composite materials [186,187]. This necessitates specialized treatments or impregnations to enhance their durability and moisture resistance, which introduces additional complexity and cost to their installation [181,188]. Moreover, the installation of natural fiber-based materials requires meticulous attention to detail, particularly in environments prone to moisture. The need for precise installation is underscored by the variability in fiber properties and their weak bonding with hydrophobic polymeric matrices, which can lead to performance inconsistencies [185,189]. The treatment processes, such as silane or alkali treatments, can improve the mechanical properties and moisture resistance of these fibers, but they also introduce additional costs and processing complexities [181,188]. Furthermore, the thermal stability of natural fibers is often lower than that of synthetic alternatives, with degradation typically occurring at temperatures above 200 °C, which can limit their application in high-temperature environments [190]. In terms of fire safety, natural fibers are generally more susceptible to ignition than synthetic materials, necessitating the incorporation of flame-retardant additives. These additives not only increase the overall cost but can also impact the environmental benefits that natural materials are often purported to offer [191]. Compliance with stringent fire protection standards in many countries poses a significant challenge, as natural insulation may not meet these requirements without substantial modifications [187]. The process of obtaining necessary certifications for natural materials can be protracted and costly, further impeding their market introduction [192]. Moreover, the current building standards and technical regulations are predominantly designed around synthetic insulation materials, creating an impediment to the adoption of natural alternatives. The transition to utilizing natural materials in construction would necessitate substantial changes to existing regulations or the acquisition of additional certifications, which can be particularly challenging in regions with rigorous building codes [192,193]. The extensive testing required to validate the properties of novel materials contributes to the financial burden, especially for smaller enterprises seeking to innovate in this domain [192]. While natural fibers offer promising environmental benefits and potential applications in insulation, their inherent properties necessitate careful consideration of treatment, installation, and compliance with safety standards. The challenges associated with moisture susceptibility, fire safety, and regulatory obstacles must be addressed to facilitate their broader acceptance and utilization in construction.

Natural fibers have garnered considerable attention in diverse applications, owing to their environmental advantages and renewable nature. Nevertheless, they exhibit several significant limitations that constrain their extensive utilization in composite materials. A primary disadvantage is their high moisture absorption capacity, which can result in the degradation of mechanical properties in the composites they reinforce. This hydrophilic characteristic leads to poor compatibility with hydrophobic polymer matrices, frequently causing issues such as delamination and void formation at the fiber–matrix interface. Moisture absorption not only affects structural integrity but also results in dimensional instability, which is critical in applications requiring precise tolerances.

To better contextualize this issue, Table 5 presents a quantitative comparison of the hygroscopic properties of selected natural and synthetic fibers. The data include the average equilibrium moisture content at 65% RH and 20 °C, the water absorption after 24 h immersion, and the percentage loss of tensile strength upon moisture exposure.

According to the data, natural fibers (flax, hemp, jute, sisal, cotton) absorb between 10% and even 25% of moisture within 24 h and can lose between 15% and 35% of their tensile strength. Their high hygroscopicity makes them more susceptible to degradation and deformation in environments with high humidity. In contrast, synthetic structural fibers such as glass, aramid, basalt, and carbon fibers have very low water absorption (less than 1%), and their mechanical properties remain virtually unchanged in humid conditions. For example, carbon fiber absorbs less than 0.2% water, which virtually eliminates the risk of moisture-related structural degradation.

At the microscopic level, the high hygroscopicity of natural fibers leads to a number of adverse phenomena in the composite structure, especially when combined with hydrophobic polymer matrices. Plant-based fibers, such as flax, hemp, and jute, contain large amounts of cellulose, hemicellulose, and lignin—highly hydrophilic compounds that easily absorb moisture from the environment. After absorbing water, the fibers swell, causing an increase in the internal stresses in the composite and weakening the interfacial adhesion by disrupting the continuity of the fiber–matrix interface [143,195,203].

One of the most commonly observed effects of this process is the microscopic detachment of fibers from the surrounding polymer matrix (known as debonding), which results in the formation of voids and local microcracks [194,204]. Mechanical loads also cause fiber pull-out from the matrix, indicating a significant weakening of interfacial adhesion. Studies using scanning electron microscopy (SEM) have shown that the interfacial areas in such composites are often irregular and porous, and after breaking tests, exposed, unattached fibers are visible [154,205].

In addition, absorbed moisture can lead to chemical degradation of cellulose components and activate biodegradation processes (e.g., by fungi and bacteria), further weakening the structural integrity of the material [206,207]. These phenomena occur particularly intensively during wetting and drying cycles, which cause structural fatigue and loss of service life of the composite.

To limit these negative effects, it is necessary to use appropriate fiber surface modification methods, such as alkalization, acetylation, silanization, or coating with hydrophobic compounds, which improve adhesion to matrices and reduce moisture absorption [201]. Another approach is to use polymer matrices with a greater polar compatibility or to design gradient interfaces in which the chemical composition between the fiber and the matrix gradually changes [144]. Studies show that appropriately modified natural fibers can exhibit significantly better durability and cohesion with the matrix under such conditions, making them a more competitive alternative to synthetic fibers.

The hydrophilic nature of natural fibers significantly limits their use in building materials, especially where these materials are exposed to moisture, rainwater, high relative humidity, and changing weather conditions. Lignocellulosic fibers exhibit high water absorption due to the presence of hydroxyl groups in the structure of cellulose and hemicellulose, which leads to swelling, biological degradation, loss of adhesion between the fiber and the matrix, and a decrease in the mechanical strength of the composite [203,208]. Outdoor applications such as facade panels, facade elements, and prefabricated elements exposed to freeze–thaw cycles are particularly sensitive to this property of fibers, where moisture can penetrate the material and accelerate its microstructural degradation. In such cases, microcracks are observed within the matrix and in the fiber–matrix interfacial zone, leading to a reduction in compressive and flexural strength [154].

The second group of applications susceptible to the negative effects of hydrophilicity is the structural elements that must demonstrate durability and stability of mechanical properties throughout their entire service life. Moisture absorption by fibers leads to a deterioration in adhesion to the polymer or cement matrix and to a reduction in the stiffness and strength of the composite, especially in humid or climatically variable environments [143]. Similar problems arise in the case of natural fiber-based insulation materials, such as hemp boards, coconut mats, or flax wool, which, by design, have a porous structure that promotes water absorption. In such cases, increased humidity not only reduces their thermal and acoustic properties but also promotes the growth of mold and microorganisms, which significantly shortens the durability of the material [209]. All of the above applications require natural fibers to be suitably modified to reduce their hydrophilicity.

In addition to moisture-related issues, natural fibers exhibit variable quality due to factors such as growing conditions, harvesting methods, and processing techniques. This inconsistency can result in unpredictable mechanical properties, thereby complicating efforts to ensure consistent performance in composite applications [184,185,210]. Furthermore, natural fibers generally exhibit lower thermal stability compared to synthetic fibers, which limits their utility in high-temperature environments [180,185,211]. This reduced thermal stability can lead to a degradation of the fibers and composite matrix, which further compromises material performance under thermal stress [191,212].

Another significant limitation is the comparatively inferior mechanical properties of natural fibers relative to synthetic alternatives, such as glass or carbon fibers. This constraint restricts their application in load-bearing structures that necessitate high strength and stiffness [186,213,214]. While chemical treatments and modifications can enhance the interfacial bond between natural fibers and polymer matrices, these processes frequently increase the complexity and cost of the manufacturing process [189,215,216]. Furthermore, the efficacy of these treatments can be variable, and they do not consistently ensure a substantial improvement in fiber–matrix interaction [10,217].

While natural fibers offer numerous advantages such as biodegradability and renewability, their high moisture absorption, quality variability, lower thermal stability, and inferior mechanical properties present significant challenges. These factors necessitate further research and development to enhance their performance and expand their utilization in composite materials. Ongoing research into the sustainability of natural fibers is increasingly focusing on innovative treatments and the development of biodegradable additives that enhance the performance of these materials while minimizing environmental impact. A significant area of investigation involves the application of environmentally benign water repellents. These treatments are designed to improve the hydrophobicity of natural fibers, thereby reducing their susceptibility to moisture absorption, which is a critical challenge due to their inherently hydrophilic nature. Treatments utilizing silane and siloxane compounds have demonstrated efficacy in enhancing the moisture resistance of natural fibers, such as flax, while maintaining their mechanical properties [218,219]. This approach not only improves the durability of the fibers in various applications but also aligns with sustainability objectives by utilizing less deleterious chemical treatments compared to conventional methods. Furthermore, the integration of biodegradable polymers with natural fibers is gaining prominence in the development of hydrophobic compositions. Biodegradable polymers, such as polylactic acid (PLA) and polyhydroxyalkanoates (PHA), are being combined with natural fibers to create composites that are both environmentally sustainable and high-performing. These composites leverage the mechanical strength of natural fibers while providing a biodegradable matrix that reduces plastic waste [160,220]. Research indicates that such combinations can lead to improved mechanical properties and thermal stability, rendering them suitable for a range of applications, from automotive to construction [123,171]. The utilization of these biodegradable matrices not only enhances the sustainability profile of the composites but also addresses the increasing concern regarding plastic pollution. In addition to these treatments, the investigation of novel natural fibers, such as those derived from the Catalpa bignonioides fruit, is also significant. This fiber exhibits low density and high biodegradability, rendering it a promising candidate for sustainable products [221]. The extraction and characterization of such fibers contribute to the diversification of raw materials available for composite production, further promoting the utilization of sustainable resources in industrial applications [222,223]. Moreover, the biofunctionalization of natural fiber-reinforced biocomposites is being investigated for applications in biomedical fields, which underscores the versatility of these materials [222]. This research emphasizes the potential of natural fibers in advanced technological applications and reinforces their role in the development of sustainable materials that can serve as alternatives to petroleum-based products. Ongoing research is actively addressing the sustainability of natural fibers through the development of environmentally benign treatments, the incorporation of biodegradable polymers, and the exploration of novel fiber sources. These efforts are essential for enhancing the performance of natural fiber composites while ensuring that they remain a viable and environmentally sustainable alternative to synthetic materials.

The typical methods of treating the surface of natural fibers to improve their compatibility with the polymer matrix primarily include silanization and alkalization. Silanization is one of the most commonly used techniques, involving the use of silane compounds that form chemical bridges between the hydroxyl groups on the fiber surface and the matrix. This increases the adhesion and moisture resistance of the composite, as well as improves the wettability and mechanical properties of the fiber–matrix interface [143,224]. Alkaline treatment, most often using sodium hydroxide (NaOH) solutions, removes the impurities, hemicellulose, and natural oils and waxes present on the fiber surface. This results in the exposure of pure cellulose, increased surface roughness, and better mechanical adhesion, which promotes more effective bonding with the matrix [150,203]. In addition to these classic methods, alternative methods of fiber surface modification, such as citric acid treatment, are gaining increasing attention. Research published by Shah et al. [225] confirms the effectiveness of this method. Citric acid introduces carboxyl groups onto the surfaces of the fibers, which can form hydrogen or covalent bonds with the matrix, especially when it is a polymer with appropriate functional groups. In addition, this acid causes a moderate depolymerization of hemicelluloses, which translates into increased surface roughness and better mechanical bonding. Treatment with citric acid further reduces the hydrophilicity of the fibers, which limits moisture absorption by the composite, increasing its dimensional stability and durability, especially in conditions of increased humidity. Silanization and alkalization remain the basic and proven methods for improving the compatibility of natural fibers with polymer matrices. Nevertheless, citric acid treatment is a promising, more environmentally friendly alternative that allows for effective surface modification without the use of strong bases or substances with higher toxicity. However, the choice of the appropriate method should be adapted to the type of fibers used, the properties of the matrix, and the requirements of the final application of the composite.

Natural fibers, despite their many advantages, have significant limitations: high moisture absorption, raw material variability, poor mechanical stability in humid conditions, low compatibility with matrices, and limited biological and thermal resistance. In response to these challenges, a growing number of researchers are focusing their efforts on four main areas. The key approach is chemical treatment of fibers, which removes non-cellulose components and changes their hydrophilicity and adhesion to the matrix. Alkalization (NaOH), silanization, acetylation, and modern methods using peroxides and potassium permanganate are commonly used [112]. For example, studies on H_2_O_2_-treated kenaf have shown a 40% increase in cellulose content and an approximately 19% increase in fiber strength [112,226]. In addition, the use of KMnO_4_ has proven effective in increasing moisture resistance and thermal stability, which is particularly important in construction applications [226]. Researchers use, among other things, advanced plasma, coronal, ultrasonic, and ozone treatments to increase the specific surface area of the fibers and remove impurities without aggressive chemical action [226,227]. Enzymatic methods (fermentation, action of enzymes, cellulolytic bacteria) effectively reduce pectin and hemicellulose while improving hydrophobicity without damaging the fiber structure [226,227]. Experiments show that composites with flax fibers treated with nanocellulose (CNC) achieve significantly higher mechanical strength and better barrier properties [228]. Scientists are designing hybrid composites (e.g., plant fibers combined with synthetic or mineral fibers), which help to balance weaker mechanical properties while maximizing environmental benefits [229]. The inclusion of nanoparticles (e.g., nanoclay, graphene, nanocellulose) improves moisture diffusion distance, increases UV stability, and enhances mechanical strength by reducing cracks [230]. For example, jute with added nanocellulose consists of a double structure that improves moisture resistance and increases the Young’s modulus by approximately 300% [231]. The growing traditional nature of natural raw materials requires a systematic approach to quality control. The use of FTIR spectroscopy, SEM microanalysis, TGA, and sorting according to fiber length and strength has become standard [97,232]. Key studies analyzing the impact of moisture have shown that, even after the water conditioning of composites with flax fibers and PLA using silica coatings, it is possible to achieve an elastic modulus retention above 43%, vs. 50% in untreated materials [232]. Studies have shown that alkalization and silanization reduce water absorption and increase the fatigue resistance of composites, improving their durability by up to 20–30% under cyclic loading and high humidity conditions [232,233]. The use of nanocoatings or nanoparticles increases resistance to moisture and UV radiation, which significantly extends the service life of building components, especially in climatically difficult areas (nanotechnology in biocomposites) [227,230]. Fermentation and enzymatic modifications are environmentally friendly compared to aggressive chemicals, while maintaining strong mechanical properties without damaging the fiber structure [226,227]. Today’s approaches to modifying natural fibers are comprehensive and integrated: from surface chemistry and physical treatments to nanoengineering and structural composite design. Such holistic strategies significantly reduce the traditional limitations of these materials, such as hygroscopicity, poor adhesion, and degradation in humid conditions, making natural fibers an increasingly viable and effective alternative in sustainable construction. The use of a combination of treatments and intelligent composite designs significantly increases their durability and stability, as confirmed by recent studies.

## 7. AI Technology: Opinion on the Question Posed in the Article

Utilizing the ChatGPT version 4.0 application, an inquiry was posed to the artificial intelligence system regarding its assessment of the potential for natural fibers to revolutionize industry and serve as an alternative to conventional materials. The use of AI Questions and Answers technology has been considered by many researchers [234,235,236,237,238,239,240,241,242,243,244,245]. The system generated several pertinent research questions. Is it feasible to entirely supplant commonly available insulation materials with natural fiber insulation? What potential obstacles might impede this transition? What modifications would be necessary to facilitate such a shift, and what are the projections for the coming decades, specifically the potential developments by 2050?

**Question 1:** *Is it feasible to entirely substitute conventional insulation materials with natural fiber alternatives?*

**Answer:** The full replacement of conventional insulation materials with natural fibers is technically feasible but practically limited by several key factors. Materials like sheep’s wool, hemp, wood fibers, and cellulose offer strong insulation performance, yet their effectiveness can vary more than synthetic options. Achieving comparable insulation may require greater thickness, and while natural fibers are breathable, they often need treatments for moisture, mold, and pest resistance. Without proper protection, they may degrade faster and be less durable than synthetics. Natural fiber insulation is often more expensive due to complex processing and lower demand, which limits economies of scale. Availability is also restricted, as many construction firms and distributors do not carry these products. Additionally, building codes and technical standards are typically designed for conventional materials like polystyrene or mineral wool, requiring natural insulations to undergo extra certification and testing. Some, like sheep’s wool, are naturally fire-resistant, but others need treatment to meet fire safety standards. Growing environmental awareness and demand for sustainable construction are driving interest and investment in natural insulation. Research and development efforts aim to improve its performance and competitiveness. However, in practice, complete replacement of synthetic materials is challenging. Hybrid solutions that combine natural and synthetic components are common to balance cost, performance, and sustainability. Natural fiber insulation is increasingly used in green and passive building projects, especially in countries like Germany, France, and Scandinavia. While substitution offers environmental and health benefits, its widespread adoption depends on technology, cost, standards, and market conditions. Thus, natural insulation is expected to complement rather than fully replace synthetic materials soon.

**Question 2:** *What barriers might be behind this?*

**Answer:** Several factors hinder the full replacement of traditional insulation materials with natural fiber alternatives. Natural fiber production is typically more resource- and labor-intensive than synthetic materials like polystyrene or polyurethane, leading to higher final costs—especially relevant in large-scale, budget-sensitive projects. Limited production scale also affects availability and pricing, and expanding output would require significant infrastructure investment. Although natural insulation can offer effective thermal performance, it often needs to be thicker than synthetic alternatives, impacting both cost and building design due to increased wall or roof dimensions. Raw materials such as flax, hemp, and wool may be limited in supply, and rising demand could strain agriculture and processing industries, increasing prices. Their availability also depends on seasonal and climatic factors, which may limit consistent large-scale production. Natural materials are more prone to biodegradation and pest infestation, especially in humid conditions, potentially reducing the lifespan compared to more durable synthetic insulation. Adoption is further slowed by limited awareness among consumers and professionals, as well as resistance within the construction industry, which is accustomed to synthetic products and would require retraining, new tools, and procedural changes. Market dominance by large synthetic insulation manufacturers and lobbying efforts can also limit opportunities for smaller producers of natural alternatives. Moreover, natural materials may require specialized installation methods or added protections like moisture barriers, increasing costs and training needs. Overall, the main barriers are economic, technical, regulatory, and supply-related. Widespread adoption of natural insulation materials will require investment in technology, policy changes, awareness campaigns, and infrastructure development. Nonetheless, the growing emphasis on sustainability in construction may gradually support its broader use.

**Question 3:** *What conditions would need to be altered to facilitate such a transformation?*

**Answer:** Raising awareness is essential. Educational campaigns should highlight the environmental, health, and energy benefits of natural insulation. Since consumer preference often favors familiar materials, promoting natural alternatives is essential. Sustainability trends and green building certifications like LEED or BREEAM can drive demand, as can the active involvement of architects and designers. Expanding the cultivation of crops like flax and hemp can increase raw material availability, lower transport costs, support local economies, and reduce carbon footprints. However, sustainable cultivation practices must be implemented to avoid ecological harm. Policy tools such as tax credits, subsidies for natural material use, and levies on synthetic insulation could accelerate adoption. Environmental regulations that limit CO_2_ emissions or require low-carbon construction practices would further support this shift. On the construction side, more efficient installation methods, worker training, and tailored tools can reduce implementation costs. Logistics must also be optimized by developing local networks for supply, storage, and transportation. Ultimately, a successful transition to natural insulation materials depends on integrated action—technological innovation, cost competitiveness, regulatory reform, political support, and market education. As sustainability awareness grows, these measures can facilitate the broad adoption of natural fibers as a viable alternative to conventional insulation.

**Question 4:** *What are the projected outcomes for the forthcoming years, specifically, what potential scenarios might materialize by 2050?*

**Answer:** By 2050, the use of natural fiber insulation materials is expected to grow significantly, driven by technological, economic, regulatory, and social trends aligned with global sustainability goals, CO_2_ reduction, and increased energy efficiency in construction. Technological advancements—particularly in biotechnology and materials engineering—are projected to improve the properties of natural fibers, enhancing their thermal and acoustic insulation, fire and moisture resistance, and durability. Innovative composites combining natural fibers with biopolymers may emerge, enabling their use in hybrid structures and broadening their applications. Mass production and growing competition are expected to reduce costs, especially as production technologies improve and the demand for raw materials, such as flax, hemp, and wool, increases. Decentralized, local sourcing of fibers may further lower transport emissions and stimulate regional economies. Natural insulation materials are likely to become increasingly cost-competitive with synthetics. Globally, stricter environmental regulations and a push for low-carbon construction will support the adoption of natural insulation. Mandatory sustainability certifications (e.g., LEED, BREEAM) and policies favoring biodegradable, low-emission materials could become widespread, especially for public and commercial buildings. Taxes on synthetic materials may also improve the market position of natural alternatives. Consumer awareness of environmental impacts is expected to rise, increasing demand for biodegradable and locally sourced insulation. The construction industry will likely shift accordingly, with more developers, architects, and contractors adopting these materials in sustainable and energy-efficient projects. By mid-century, new sources of natural fibers—possibly derived from fast-growing plants or lab-engineered biomaterials—may offer greater resilience and performance. Biotechnology and genetic engineering could enable the development of naturally fire-resistant fibers, reducing the need for chemical additives. The circular economy is projected to shape the construction sector, favoring materials that are recyclable or biodegradable. Business models integrating production, installation, and end-of-life recovery of insulation materials are expected to become common, reducing waste and costs. By 2050, natural fiber insulation is expected to become a mainstream solution in sustainable construction, supported by innovation, regulation, and environmental awareness. These materials are likely to play a key role in addressing climate challenges while promoting a low-carbon, circular building economy.

**Question 5:** *Considering all of the above factors, do you think natural fiber insulation will replace commonly available insulation materials, do we need such a change, and when might such a change occur?*

**Answer:** Replacing common insulation materials with natural fibers is theoretically possible but will be a gradual process. Change is necessary because conventional materials (rock wool, polystyrene, polyurethane foam) come from fossil resources and have a high carbon footprint, and recycling is difficult. Natural insulations reduce CO_2_ emissions, are biodegradable or easier to recycle, and pose fewer health risks, improving indoor air quality. The change is viable if natural materials can compete in performance (insulation, fire, and moisture resistance) and cost. Government support through regulations and stakeholder awareness is essential. Until 2030, natural fibers will remain niche, mainly in passive and sustainable buildings. Between 2030 and 2040, their market share may grow to 10–20%, and by 2040–2050, they could become dominant if climate neutrality is achieved. A complete replacement of synthetics is unlikely due to specific technical demands. A complementary use of materials is expected: natural fibers being dominant in sustainable and residential construction, and synthetics being used where special properties are required. The transition requires research, government backing, and public acceptance. A full market transformation might happen by 2050, but the coexistence of natural and improved synthetic materials is more probable, enabling more sustainable construction.

## 8. Summary: A Critical Analysis of the Potential for Natural Fibers to Revolutionize Material Science and Serve as Alternatives to Conventional Materials

### 8.1. Critical Analysis of Properties

The analysis of the properties of traditional insulation materials and natural fibers covers various aspects, such as thermal insulation, acoustic properties, durability, environmental impact, and cost. The most commonly used materials, such as mineral wool, expanded polystyrene (EPS), or polyurethane (PUR) foam, have good insulating properties. The thermal conductivity coefficients (λ) are 0.031–0.040 W/(m × K) for polystyrene, 0.030–0.045 W/(m × K) for mineral wool, and 0.020–0.035 W/(m × K) for PUR foam. Fibers such as cotton, sheep’s wool, hemp, flax, cellulose, or coir fibers have higher thermal conductivity coefficients: 0.035–0.045 W/(m × K) for sheep’s wool, 0.037–0.041 W/(m × K) for cellulose, and 0.040–0.045 W/(m × K) for hemp. Natural fibers are slightly less efficient as thermal insulation than most synthetics. Mineral wool stands out for its excellent acoustic properties, attenuating noise at 40-60 dB. EPS and PUR have a lower efficiency in this range. EPS is better at impact sound attenuation, while air insulation is low (approx. 15–20 dB). The open-cell version of PUR achieves better air insulation (25–30 dB) and comparable impact sound attenuation. Materials such as sheep’s wool or cellulose have sound insulation properties, similar to mineral wool, thanks to their structure, which allows sound absorption. They can achieve attenuation of 40–50 dB. Polystyrene and polyurethane foam are resistant to moisture but lose their properties when exposed to UV or high temperatures. Mineral wool is durable but sensitive to humidity, requiring additional moisture barriers. Materials such as sheep’s wool and cellulose are susceptible to mold and fungal growth in high-humidity conditions. To prevent this, they require impregnation (e.g., with boron salts). Their durability is lower compared to synthetics if they are not properly treated [246,247,248,249,250,251,252]. The production of polystyrene and PUR foam generates significant CO_2_ emissions and waste that is difficult to dispose of. They are based on non-renewable raw materials (oil) and have a high carbon footprint. CO_2_ emissions for 10 cm-thick polystyrene foam are 5–7.5 kg CO_2_/m^2^, while energy consumption can be 200–500 MJ/m^3^. The CO_2_ for 5 cm PUR foam is 8–21 kg CO_2_/m^2^, while the energy consumption is 500–800 MJ/m^3^. Natural fibers are renewable, biodegradable materials and have a low carbon footprint. However, production requires energy and water, and impregnation can affect their environmental performance. Nevertheless, they have a lower environmental impact than synthetics throughout their life cycle. Natural fibers have a lower carbon footprint and lifecycle energy consumption but require a higher volume for comparable insulation performance. CO_2_ emissions are 0.2–1 kg CO_2_/kg material, while energy consumption is 50–200 MJ/m^3^ [253,254,255,256,257]. Traditional insulation materials are mass-produced and relatively inexpensive: polystyrene foam: 37.5–50 USD/m^3^ and mineral wool: 20–37.5 USD/m^3^. Natural fibers tend to be more expensive due to smaller-scale production and impregnation costs: cellulose: 30–50 USD/m^3^ and sheep’s wool: 50–100 USD/m^3^. Mineral wool can cause skin and respiratory irritation during installation. Extruded polystyrene and PUR can emit volatile organic compounds (VOCs) during use. They are safe for the health of users and do not irritate. The risk only arises in the context of mold if the materials are poorly impregnated or used in a humid environment. Traditional insulation materials offer better thermal performance than natural fibers. Natural fibers have an advantage due to their renewability and biodegradability. Traditional materials are more cost-effective for large projects. Natural fibers perform better where health and ecology are a priority. The choice of material depends on the specific requirements of the project, such as budget, ecological objectives, and environmental conditions [258,259,260].

### 8.2. Advantages of Natural Fibers as Insulation Materials

**Sustainability and renewability:** Natural fibers are renewable resources, which present a significant advantage over synthetic materials derived from fossil-based raw materials. The utilization of natural fiber thermal insulation can potentially result in a reduction of CO_2_ emissions by up to 10% [261]. Effective building insulation incorporating natural fibers can mitigate more than several times the carbon dioxide associated with the production and disposal of the material, thereby contributing substantially to the reduction of greenhouse gas emissions [262]. The production process of natural fibers consumes approximately 60% less energy compared to that of synthetic fibers, consequently resulting in decreased emissions of harmful gases into the atmosphere and a reduced carbon footprint [263].

**Biodegradability:** Natural fibers are readily biodegradable, mitigating the issue of long-term waste accumulation. Synthetic materials such as extruded polystyrene can persist in the environment for centuries, contributing to environmental pollution.

**Health safety:** Natural fibers exhibit lower toxicity when combusted and do not release harmful substances during utilization, rendering them more favorable for human health.

**Good insulation properties:** Numerous natural fibers, particularly sheep’s wool and hemp, exhibit significant thermal retention and acoustic insulation properties, approximating the efficacy of conventional synthetic materials.

### 8.3. Limitations of Natural Fibers

Natural fibers are extensively utilized in diverse industries, particularly in textiles, composites, and construction. Notwithstanding their numerous advantages, such as biodegradability, renewability, and low energy consumption during production, natural fibers also exhibit several disadvantages that warrant consideration. This article aims to elucidate three significant disadvantages: flammability, regional accessibility, and transportation challenges [100,172,264,265,266,267,268,269,270].

#### 8.3.1. Flammability: Flammability Mechanisms and Strategies to Address the Problem

One of the most significant disadvantages of natural fibers is their flammability. Natural fibers such as cotton, wool, and flax are organic materials that ignite when exposed to open flames or elevated temperatures. This characteristic presents a substantial safety hazard, particularly in applications where fire resistance is essential, such as building materials, automotive interiors, and upholstery. The flammability of natural fibers can be attributed to their chemical composition, which primarily comprises cellulose, hemicellulose, and lignin. These organic compounds are highly combustible, potentially leading to rapid fire propagation. The flash point of natural fibers is frequently lower than that of synthetic fibers, rendering them more susceptible to ignition. Various treatments and additives can be employed to mitigate flammability. Flame retardants can be applied to natural fibers to enhance their fire resistance. However, the utilization of chemical treatments raises concerns regarding environmental impact and potential toxicity. Consequently, the development of environmentally benign flame retardants remains an active area of research.

#### 8.3.2. Availability in Selected Regions, Regional Differences, and Economic Implications

The geographical availability of natural fibers is often limited, which can restrict their utilization in certain regions. The production of natural fibers is highly dependent upon climatic conditions, soil types, and agricultural practices. For instance, cotton flourishes in warm climates, while flax is predominantly cultivated in regions with temperate climates. The concentration of natural fiber production in specific areas can result in supply chain vulnerabilities. For example, nations such as India and China are major producers of cotton, while Europe is renowned for its flax production. Regions lacking adequate agricultural conditions may encounter challenges in sourcing these materials, leading to increased costs and dependence on imports. The limited availability of natural fibers in some regions can also have economic implications. Local industries may struggle to compete with regions that possess abundant natural fiber resources, potentially resulting in job losses and economic decline in fiber-deficient areas. This disparity underscores the necessity for diversification in fiber sourcing and the exploration of alternative materials.

#### 8.3.3. Transportation Issues, Logistics and Costs, and Environmental Impact

The transportation of natural fibers presents significant logistical challenges that can impact their overall sustainability and economic viability. The inherent bulkiness and weight of natural fibers can result in elevated transportation costs, particularly when fibers are sourced from remote agricultural regions. The transportation of natural fibers often necessitates specialized handling and storage protocols to mitigate damage and degradation. For instance, fibers such as jute and sisal exhibit susceptibility to moisture, which can precipitate mold growth and quality deterioration during transit. Consequently, the expenses associated with transportation can substantially influence the final price of products derived from natural fibers. Furthermore, the environmental implications of transporting natural fibers warrant consideration. The carbon footprint attributable to long-distance transportation has the potential to negate some of the environmental advantages of natural fibers. This issue underscores the significance of local sourcing strategies and the development of regional supply chains to minimize transportation-related emissions.

To sum up, natural fibers possess numerous advantages. However, their limitations must be critically evaluated, including flammability, regional availability, and logistical challenges. Addressing these concerns necessitates a comprehensive approach, encompassing the development of fire-resistant technologies, diversification of fiber sources, and optimization of transportation systems. Subsequent research should prioritize sustainable methodologies that mitigate these limitations while maximizing the potential benefits of natural fibers across various applications.

In recent years, natural fibers have gained prominence as a potential alternative to synthetic insulation materials, such as polystyrene, mineral wool, and polyurethane foam. The consideration of their role in revolutionizing the insulation industry is predicated on increasing environmental awareness and the demand for sustainable, environmentally friendly solutions. However, certain limitations may impede their widespread adoption. The following presents a critical analysis of the topic.

Other limitations of natural fibers include:

- Production costs and availability: While natural fibers are renewable resources, their large-scale production can be costly and resource-intensive, particularly in terms of water and land usage. In comparison to synthetic materials, which can be manufactured cost-effectively, natural fibers generally incur higher production expenses;
- Limited durability: Natural fibers exhibit increased susceptibility to moisture, mold, fungi, and pests. They may undergo more rapid decomposition or experience a reduction in insulating properties under specific conditions, rendering them less advantageous in comparison to more durable synthetic alternatives;
- Mechanical properties: While natural fibers exhibit favorable insulating properties, their mechanical strength is frequently inferior to that of synthetic materials. This limitation restricts their application in certain contexts where high compressive strength or dynamic loads are necessary;
- Scale and logistics: Large-scale production of natural fibers that could meet global demands would necessitate substantial modifications in agricultural and industrial practices. The extensive cultivation of hemp, flax, or jute presents considerable logistical challenges, encompassing raw material transportation, processing, and storage.

### 8.4. The Challenges of Adaptation

**Regulations and standards:** Insulating materials used in construction must comply with strict building codes and regulations that cover several key performance areas, including thermal insulation efficiency, fire resistance, moisture resistance, and long-term durability. These standards are essential to ensure the safety, energy efficiency, and longevity of buildings. However, natural fiber-based insulating materials, such as those derived from hemp, flax, jute, or wool, often struggle to meet these rigorous requirements in their raw form. In many cases, they require chemical treatments or synthetic additives to enhance properties like fire resistance or resistance to mold and pests. Unfortunately, these additional treatments can diminish the environmental advantages that make natural fibers appealing in the first place, such as their biodegradability and low carbon footprint. The challenge, therefore, lies in striking a balance between meeting regulatory standards and preserving the ecological benefits of natural insulation materials [118,271,272,273,274].

**Public and industry opinion:** Conventional insulation materials, such as mineral wool, fiberglass, and expanded polystyrene, have long dominated the construction market. Their widespread use is supported by decades of empirical testing, performance data, and regulatory approvals. As a result, they are considered reliable and cost-effective by both professionals and consumers. Introducing natural fiber insulation into this well-established market faces significant resistance. Many builders and developers are hesitant to adopt alternative materials that may be perceived as less proven, more expensive, or more difficult to source. Additionally, public awareness of natural insulation options remains relatively low, which further limits demand and commercial momentum. Overcoming these obstacles requires not only further technical development of natural materials but also efforts in education, marketing, and policy support to shift perceptions and encourage adoption [275,276,277,278,279].

### 8.5. Potential for the Future

Natural fibers may potentially serve as a significant component in sustainable construction practices of the future, particularly in nations where the production of these raw materials is already well-established. Processing technologies for natural fibers can potentially advance, enhancing their durability and insulating properties. The advancement of composite technologies, wherein natural fibers are integrated with other materials, could potentially expand the scope of their application.

Nevertheless, for these natural fibers to significantly impact the field of insulation, they must address challenges related to cost, performance, and scalability. Currently, natural fibers remain a specialized alternative that may exhibit superior performance compared to synthetic materials in certain applications. However, it is unlikely that they will entirely supplant conventional materials on a large scale soon. While natural fibers possess numerous advantages that render them a promising alternative to conventional insulation materials, their potential to significantly impact the market is constrained by technical and economic challenges. As processing technology progresses and emphasis on environmentally sustainable solutions increases, natural fibers may assume a more prominent role. However, widespread adoption will necessitate substantial modifications in manufacturing infrastructure and industrial methodologies.

An innovative solution that could be implemented in the future, particularly in the prefabrication industry, is the utilization of locally available natural fibers encapsulated in a concrete monolithic matrix. This approach addresses the issues of flammability and transportation. The incorporation of natural fibers in the prefabrication industry, combined with geopolymers, represents a novel approach with the potential to revolutionize the production of building materials. The primary concept involves encapsulating locally sourced natural fibers, such as flax or hemp, within a geopolymer matrix, thereby combining the environmental advantages of fibers with the superior mechanical properties of geopolymers. This technology offers benefits in terms of sustainable construction and production efficiency. Geopolymers are advanced binder materials that provide an environmentally favorable alternative to traditional binders such as Portland cement. They are synthesized from silica- and aluminum-rich raw materials, including fly ash, metakaolin, or volcanic aggregate dust, which are chemically activated using alkaline solutions. Compared to Portland cement, the geopolymer production process generates a significantly reduced carbon footprint. Geopolymers exhibit high resistance to chemical agents, such as acids and salts, as well as to elevated temperatures. Additionally, they form robust and durable structures, rendering them suitable for construction applications, particularly in extreme environments. The incorporation of natural fibers into a geopolymer matrix yields significant advantages through the synergistic combination of these materials. While natural fibers are inherently less robust than synthetic alternatives, they can substantially enhance the tensile and compressive strength of geopolymers. Consequently, prefabricated structures composed of this composite material can be lightweight yet possess sufficient strength for their intended applications. A significant advantage of natural fibers is their widespread availability in various geographical regions. In Europe, for instance, hemp and flax are prevalent options. The utilization of locally sourced fibers can substantially reduce transportation costs and minimize the carbon footprint associated with the entire production process. Natural fibers exhibit great insulating properties, and when combined with geopolymers, they can yield prefabricated materials with enhanced thermal insulation characteristics. This enables their use as substitutes for more environmentally detrimental insulation materials, such as polyurethane foams and mineral wool. The natural fibers embedded within the geopolymer matrix can be molded into diverse configurations, facilitating the production of prefabricated building components with complex architectural forms, including panels, walls, and structural elements. The geopolymer matrix functions as a binding agent, allowing for a facile molding of the material during the production phase. Prefabrication, the process of manufacturing building components under controlled conditions, offers numerous advantages, including precision manufacturing, conservation of raw materials, and reduced on-site construction time.

The utilization of local natural fibers in prefabrication, in conjunction with geopolymers, aligns with the principles of circular economy and sustainability, as prefabricated elements can be produced with minimal material waste, and the application of geopolymers and natural fibers can substantially reduce CO_2_ emissions. The use of local raw material resources diminishes the necessity for long-distance transportation, further reducing transportation emissions and supporting local economies. Natural fibers are biodegradable, and geopolymer components possess potential for recycling. These materials can be reprocessed in the future, thereby reducing environmental impact. While the combination of natural fibers and geopolymers presents numerous advantages, several challenges exist in commercializing this technology. Natural fibers exhibit diverse physical and chemical properties that may affect the performance of the final product. Additional research is required to determine optimal methods for modifying the fibers to enhance their adhesion to the geopolymer matrix and increase their strength. Reliance on locally available raw materials can be both advantageous and challenging, as the diversity of natural fibers globally may lead to variability in the quality and availability of final products. Although geopolymer binders are more environmentally friendly than cement, their large-scale production remains relatively novel, and costs may exceed those of traditional building materials. Prefabricated products composed of natural fibers and geopolymers have potential applications in a wide range of industries, including housing, infrastructure, industrial construction, and reconstruction and renovation projects. This technology may be particularly effective in regions with a high availability of natural fibers and in locations where green building standards are prioritized, such as in Europe. Future developments may include specialized geopolymer composites incorporating natural fibers that can compete with traditional materials in the prefabrication market, offering superior environmental and insulation properties. The combination of natural fibers with geopolymers in prefabrication represents an innovative and environmentally conscious approach to the production of building materials. While technical challenges persist, this solution has the potential to significantly impact sustainable construction and benefit both the environment and the prefabrication industry [280,281].

### 8.6. Summary: Answer to the Main Question

When it comes to the question of whether natural fibers can revolutionize the insulation industry and become a viable alternative to popular synthetic materials, the answer is complex. Although it does not currently seem possible to completely replace synthetic materials with natural fibers, their role in modern construction is gradually growing. Natural fibers have many advantages, such as renewable raw materials, a low carbon footprint, and good thermal insulation properties, so it is worth considering their use where it is technologically and economically justified. Certain challenges, such as durability, fire resistance, and susceptibility to moisture, mean that natural materials require further development, including appropriate impregnation and protection techniques. On the other hand, synthetic materials such as polystyrene, polyurethane foams, and mineral wool have been optimized for decades in terms of durability, safety, and energy efficiency. Therefore, their share in mass construction remains dominant. However, growing interest in sustainable development and the requirements of the European Green Deal are stimulating the development of alternative materials, including natural insulators. Although they are currently more expensive and less widespread, research into improving them may increase their competitiveness in the future. At the same time, it should be remembered that the development of insulation materials also includes modern solutions, such as composites, vacuum panels, and aerogels, which offer very high energy efficiency. Natural fibers can be a valuable addition to this spectrum, especially in green and low-carbon construction. In summary, instead of thinking in terms of complete replacement, a complementary approach seems more appropriate, in which both natural and synthetic materials have their place—depending upon the application, requirements, and environmental context. Reasonable compromises between durability, safety, and ecology are the key to sustainable development in the construction industry.

### 8.7. The Future of Natural Materials

When considering insulation materials based on natural fibers and synthetic composites, it is important to identify the trade-offs associated with their durability, performance, and use in different environmental conditions. Natural fibers are biodegradable, making them environmentally friendly. With appropriate impregnation and modification processes, the durability of these materials can be increased. They are more susceptible to degradation by moisture, mold, insects, and UV radiation. The need for protective measures can increase costs and environmental impact. Synthetic composites have high resistance to moisture, mold, and weathering, making them more durable for long-term applications. However, they are more difficult to recycle, and their degradation can lead to the formation of microplastics, which is an environmental concern. Natural fibers have good thermal insulation properties due to their porous structure. In addition, their ability to absorb moisture can stabilize the microclimate inside buildings. High moisture absorption can lead to a reduction in their insulating properties if the materials are not properly protected. Synthetic composites maintain their high insulating performance even under harsh environmental conditions, such as moisture or extreme temperatures. The performance of these materials is often associated with a higher carbon footprint during production. Natural fibers are often produced from renewable raw materials, such as hemp, flax, or sheep’s wool, which can reduce raw material costs in regions where they are readily available. The cost of processing and modification to meet utility requirements can be high. In addition, availability may be limited in some regions. Synthetic composites are widely available and can be produced in large quantities at relatively low unit costs. However, the environmental costs associated with production and subsequent disposal are significantly higher than for natural materials. Natural fibers are biodegradable and have a low carbon footprint during production, making these materials more sustainable. However, their high water absorption may require the use of protective chemicals, potentially reducing their environmental friendliness. Synthetic composites are characterized by durability and strength, which can reduce the frequency of material replacement, reducing their environmental impact to some extent. However, low biodegradability and difficulty in recycling pose a major environmental problem. The choice between natural fibers and synthetic composites depends on the user’s priorities: whether he or she prioritizes environmental performance or performance and durability in harsh environments. Further research should focus on optimizing natural materials by improving their durability and resilience without negative environmental impact, as well as developing more sustainable technologies for synthetic composites.

Natural fibers have a wide potential for industrial applications, especially in the context of the drive toward more sustainable and eco-friendly solutions. However, their practical use and scalability come with specific technological and economic challenges. Fibers such as flax, hemp, sheep’s wool, and coir are used as natural insulation materials in residential and commercial buildings. They can be used on walls, roofs, and floors. Natural fiber materials are used as lightweight, strong composite panels for wall construction or as replacements for OSB and MDF. In the automotive industry, they are used in the manufacture of interior panels, covers, seats, and dashboards, replacing synthetic fibers such as fiberglass. The lower weight of the components translates into fuel savings, and the biodegradability of the materials reduces their environmental impact at the end of their life. Natural fibers such as jute, flax, and cellulose are used in the production of biodegradable packaging, bags, and sacks. The increase in demand for eco-friendly solutions in the FMCG industry is supporting the growth of this segment. Natural fibers are used in the production of specialty fabrics, such as filters, geotextiles, and protective materials used in the construction, agriculture, and civil engineering industries. Cellulose derived from plant fibers, such as hemp and flax, provides the raw material for the production of high-quality paper, including technical, banknote, and filter paper. In terms of scalability advantages, natural fibers such as hemp, flax, and jute can be grown under a variety of climatic conditions, ensuring a wide availability of raw materials. Basic fiber processing, such as cutting, pressing, and impregnation, is relatively simple and can be scaled up using existing technologies. Waste from crops, such as maize stalks or rice husks, can be efficiently processed into fibers. Natural fibers are characterized by a diversity of physical properties depending on the type of plant, growing conditions, and processing methods, which makes it difficult to standardize products. Natural fibers are susceptible to degradation (e.g., by moisture and mold), requiring additional treatments such as impregnation, which increase production costs. The cultivation of crops providing natural fibers is dependent upon seasons and weather conditions, which can limit the availability of raw materials at certain times. The use of fiber crops for industry may compete with their use for food or other raw materials, which may limit their scalability. Potential solutions include investing in more advanced processing technologies that can increase efficiency and reduce the cost of large-scale natural fiber production. Combining natural fibers with synthetic fibers can improve the durability and performance of materials while reducing their environmental impact. Building processing plants close to growing areas can reduce transport costs and increase the profitability of production. Natural fibers have significant potential in many industries, especially in construction, automotive, and packaging. However, their scalability is limited by technological, economic, and environmental challenges. The implementation of modern technologies, the development of hybrid materials, and the standardization of processing processes can significantly increase their use in industrial settings and contribute to the large-scale popularization of these eco-friendly solutions.

With the growing interest in environmentally friendly materials, natural fibers offer extensive opportunities for research and innovation. Future research directions should focus on solving current limitations and exploring new applications to enhance the performance and sustainability of these materials. Research into hybrid composites should be pursued. Optimization of component ratios must be considered. Research should be carried out on the ideal ratio of natural-to-synthetic fibers for optimal mechanical and thermal properties. An adhesion analysis between natural fibers and polymer matrix, to increase mechanical strength, is needed. The use of biodegradable polymers (e.g., PLA, PHA) in combination with natural fibers will create completely eco-friendly composites. This will lead to the development of lightweight, high-strength materials for automotive, aerospace, and construction applications. For natural fibers to compete with synthetic materials, it is necessary to have a thorough understanding of their performance over the long term, especially under varying environmental conditions. Research areas to be addressed include moisture and mold resistance (analyzing the long-term effects of moisture on the structure and insulating properties of fibers), biodegradation (monitoring the biodegradation process of natural fibers and its impact on durability), UV and temperature resistance (investigating the stability of fibers under intense sunlight and high or low temperatures), and cyclic testing (analyzing the behavior of materials under cyclic mechanical and temperature loads). To modify the fibers, research should be carried out on their treatment to increase their moisture resistance and improve their mechanical properties. The integration of nanomaterials (e.g., silicon nanoparticles, graphene) into the structure of natural fibers can increase their strength and functionality. The development of industrial processes will enable the mass production of natural fibers and composites in an economically viable manner. The introduction of new methods, such as 3D printing using natural fibers, will allow for the automation of production. Research into the full life cycle of natural fiber materials, from raw material cultivation to disposal, to assess their environmental impact compared to synthetic materials, will influence the sustainability of these materials. It is also necessary to develop technologies to effectively recycle composites containing natural and synthetic fibers. There is also a need to develop technologies that allow agricultural waste, such as rice husks, corn stalks, or straw, to be transformed into high-quality insulating fibers.

Future research into natural fibers should focus on:

- Development of hybrid composites with improved performance and durability;
- Long-term analysis of performance under varying environmental conditions;
- Implementation of new processing technologies and recycling methods;
- Exploration of eco-friendly geopolymer matrix in association with fibers;

These directions can contribute to the creation of materials that combine the advantages of environmental friendliness with high performance, opening up new opportunities in construction, transport, and other industries.

## Figures and Tables

**Table 1:** Comparison of house construction methods [39,40,41,42,43,44,45,46,47,48,49,50,51,52].

Method	Main Materials	Geographic Reach	Advantages	Disadvantages	Historical/Modern Examples
Wooden construction	Wood	Northern Europe, North America, Asia	Lightweight, flexible, easy to process	Moisture, susceptibility to fire	Log houses, frame houses
Masonry construction	Brick, stone	Europe, Middle East, Asia	Durability, thermal insulation	Weight, low flexibility	Traditional brick houses
Earthworks	Earth, clay	Latin America, Africa, Asia	Low costs, ecology, insulation	Sensitive to moisture, requires maintenance	Adobe, rammed earth
Concrete construction	Concrete, reinforced concrete	Globally	Strength, prefabrication	High carbon footprint, heavy weight	Skyscrapers, prefabricated houses
Natural construction	Straw, bamboo, hemp	Europe, Asia, South America	Ecology, insulation	Requires protection against moisture and fire	Straw houses, bamboo houses
Prefabricated and modular construction	Various (wood, concrete)	Globally	Speed, quality control	Limited customization	Prefabricated housing estates
Stone construction	Stone	Ancient cultures	Durability, monumentality	Lack of flexibility, weight	Pyramids, Stonehenge
Clay and wicker construction	Clay, wood, straw	Europe, Africa, Asia	Availability of materials, insulation	Maintenance, sensitivity to conditions	Wattle and daub

**Table 2:** Comparison of natural fiber processing methods [110,111,112,113,114,115,116].

Method	Advantages	Disadvantages	Application
Alkalization	Better adhesion to the matrix	Possibility of fiber degradation	Composites with cement and polymers
Acetylation	Reduced water absorption	Expensive	Wood-based composites
Silanization	Good compatibility with resins	A complex process	Polymer laminates
Fermentation	Eco-friendly, natural	Long process time	Composites of bioactive materials

**Table 3:** List of composites with natural fibers and their parameters (examples) [117,118,119,120,121,122].

Matrix	Fiber Type	Flexural Strength [MPa]	Thermal Conductivity [W/m·K]	Application
PLA	Flax	60–90	0.04–0.07	Wall panels
Cement	Hemp	5–10	0.10–0.15	Wall blocks, plaster
Bio-resin	Coconut	15–30	0.05–0.08	Thermal insulation
Epoxy	Jute	40–80	0.03–0.06	Facades, roofing

**Table 4:** Comparison of mechanical, thermal, and environmental properties of natural and synthetic fibers [129,141,142,143,144,145,146,147,148,149,150,151,152,153,154,155,156,157,158,159].

Feature	Flax	Hemp	Cotton	Coconut	Jute	Sisal	Carbon	Aramid (Kevlar)	Glass (E-glass)	Basalt
**Tensile strength** **[MPa]**	500–900	550–900	287–597	175–220	400–800	500–700	3000–6000	3000–3600	2000–3500	2000–4800
**Modulus of elasticity [GPa]**	27–80	30–70	5.5–12.6	4–6	20–55	9–22	230–600	70–130	70–85	85–95
**Elongation at break** **[%]**	1.2–3.2	1.6–2.8	3–10	15–45	1.5–2.0	2–3	0.5–1.5	2.5–4.5	2.5–4.8	3.1–4.5
**Density** **[g/cm^3^]**	1.4–1.5	1.47	1.5–1.6	1.2–1.3	1.3–1.5	1.45	1.75–2.00	1.44	2.5–2.6	2.7–2.9
**Thermal conductivity [W/m·K]**	~0.04–0.06	~0.04–0.06	~0.03–0.05	~0.045–0.06	~0.04–0.05	~0.04–0.05	5–20	0.04–0.05	0.9–1.2	0.03–0.038
**Melting/degradation point [°C]**	~200	~200	~210	~200	~200	~200	>3500 (don’t melt)	~500 (degradation)	~850	~1450
**Biodegradability**	Yes	Yes	Yes	Yes	Yes	Yes	No	No	No	No
**Raw material renewability**	Yes	Yes	Yes	Yes	Yes	Yes	No	No	No	No
**Environmental impact (production)**	Low	Low	Medium-high	Low	Low	Low	Very high	High	High	Medium
**Recyclability**	Limited	Limited	Limited	Limited	Limited	Limited	Yes (partial)	Yes	Yes	Yes

**Table 5:** Comparison of moisture-related properties of selected natural and synthetic fibers [144,154,194,195,196,197,198,199,200,201,202].

Fiber Type	Equilibrium Moisture Content (%)	Water Absorption After 24 h (%)	Decrease in Tensile Strength (%)
Flax	7–12	10–15	15–25
Hemp	8–10	10–14	10–20
Jute	12–13	15–20	20–30
Coconut	8–10	8–12	10–18
Cotton	7–9	15–25	20–35
Sisal	10–11	12–18	15–30
Glass fiber	~0.1	<0.5	~0
Aramid (Kevlar)	3–7	4–6	<5
Basalt	~0.2	<1	<2
Polipropylen (PP)	~0.05	<0.5	negligible
Carbon	<0.1	<0.2	<2

## Data Availability

No new data were created or analyzed in this study. Data sharing is not applicable to this article.
